# Loss of HER2 in breast cancer: biological mechanisms and technical pitfalls

**DOI:** 10.20517/cdr.2022.55

**Published:** 2022-10-20

**Authors:** Stefania Morganti, Mariia Ivanova, Emanuela Ferraro, Liliana Ascione, Grazia Vivanet, Giuseppina Bonizzi, Giuseppe Curigliano, Nicola Fusco, Carmen Criscitiello

**Affiliations:** ^1^Division of Early Drug Development for Innovative Therapies, IEO, European Institute of Oncology IRCCS, Milan 20144, Italy.; ^2^Department of Oncology and Haemato-Oncology, University of Milano, Milan 20122, Italy.; ^3^Breast Oncology Center, Dana-Farber Cancer Institute, Boston, MA 02215, USA; Harvard Medical School, Boston, MA 02215, USA.; ^4^Biobank for Translational and Digital Medicine Unit, IEO, European Institute of Oncology IRCCS, Milan 20144, Italy.; ^5^Division of Pathology, IEO, European Institute of Oncology IRCCS, Milan 20144, Italy.; ^6^Breast Medicine Service, Department of Medicine, Memorial Sloan Kettering Cancer Center, New York, NY 10065, USA.

**Keywords:** HER2 loss, breast cancer, subtype switch, tumor heterogeneity, clonal selection, HER2 downregulation, technical pitfalls

## Abstract

Loss of HER2 in previously HER2-positive breast tumors is not rare, occurring in up to 50% of breast cancers; however, clinical research and practice underestimate this issue. Many studies have reported the loss of HER2 after neoadjuvant therapy and at metastatic relapse and identified clinicopathological variables more frequently associated with this event. Nevertheless, the biological mechanisms underlying HER2 loss are still poorly understood. HER2 downregulation, intratumoral heterogeneity, clonal selection, and true subtype switch have been suggested as potential causes of HER2 loss, but translational studies specifically investigating the biology behind HER2 loss are virtually absent. On the other side, technical pitfalls may justify HER2 loss in some of these samples. The best treatment strategy for patients with HER2 loss is currently unknown. Considering the prevalence of this phenomenon and its apparent correlation with worse outcomes, we believe that correlative studies specifically addressing HER2 loss are warranted.

## INTRODUCTION

Human epidermal growth factor receptor-2 (HER2)-positive breast cancer (BC) accounts for 15%-20% of breast carcinomas^[1]^. Although originally associated with a poor prognosis, the advent of anti-HER2 agents has dramatically changed the natural history of HER2-positive BC. Most patients with early-stage disease are now cured following (neo)adjuvant therapy^[2,3]^, and many patients with advanced disease live many years after diagnosis^[4,5]^. This unprecedented success is due to the development of numerous highly effective HER2-targeting agents capable of inhibiting the HER2 signaling pathway, which is the main driver of cancer cell proliferation and progression for this breast cancer subtype^[6]^. Indeed, HER2 status is not only a prognostic biomarker but also a strong predictor of response to HER2-targeted therapies^[6,7]^. 

According to 2018 American Society of Clinical Oncology (ASCO)/College of American Pathologists (CAP) guidelines, HER2 status is assessed by both immunohistochemistry (IHC) and *in situ* hybridization (ISH)^[8]^. The former describes the expression level of the HER2 protein on the tumor cell membrane, while the latter reflects the amplification of the *ERBB2* gene at the nuclear level. The results of IHC and ISH are consistent in most BCs. However, some cases may instead be “borderline” or equivocal, and they are codified in specific groups by ASCO/CAP guidelines^[8]^.

**Figure 1:**
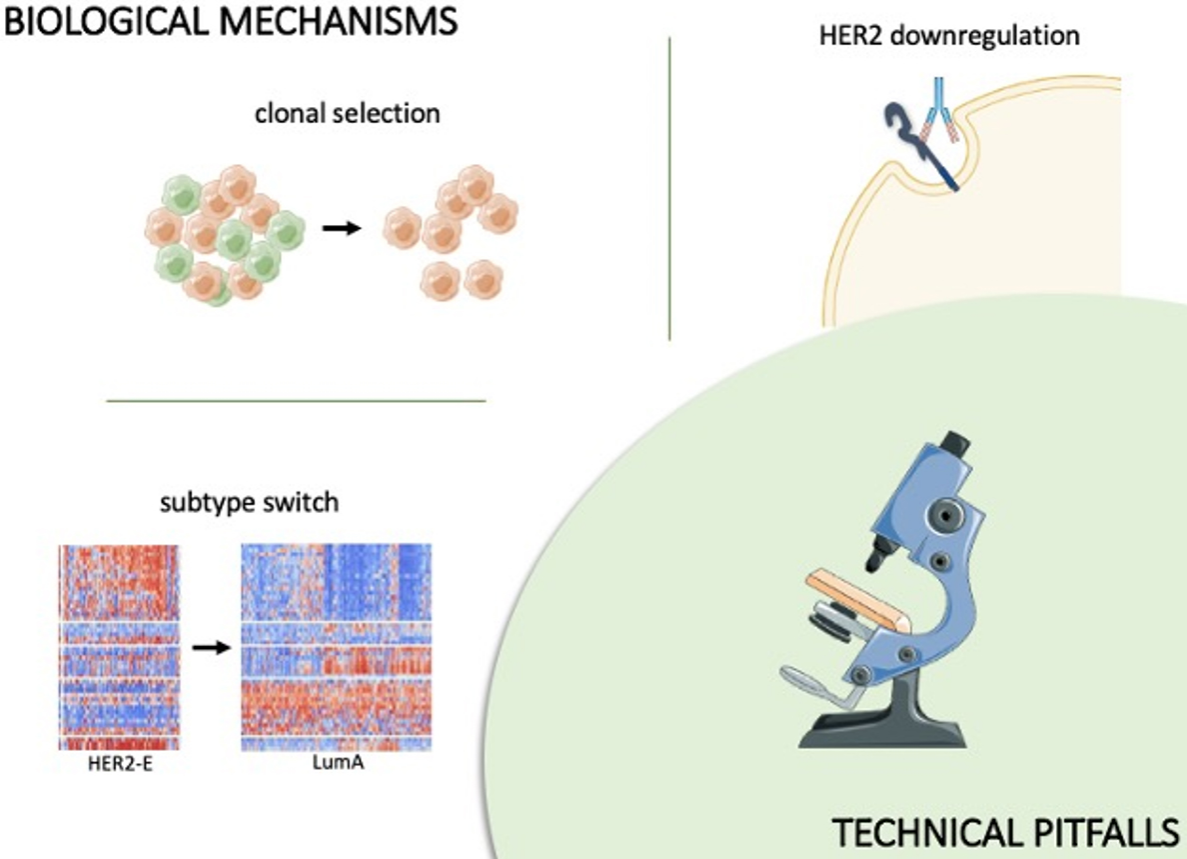
HER2 downregulation, subtype switch, and clonal selection are biological mechanisms potentially responsible for HER2 loss. In parallel, technical pitfalls can cause false-negative results. Figure partly generated using Servier Medical Art, provided by Servier, licensed under a Creative Commons Attribution 3.0 unported license and adapted from Mathews *et al*.^[13]^ (http://creativecommons.org/licenses/by/4.0/). HER2: Human epidermal growth factor receptor-2.

Discordance between HER2 status in paired samples has been described previously in both early and advanced setting^[9,10]^. Specifically, the loss of HER2 is defined as a negative HER2 status in a previously HER2-positive tumor (i.e., a change from a 3+ IHC score or 2+/ISH-amplified to an IHC 0-1+ or 2+/ISH-negative), implying that the residual tumor no longer has HER2 overexpression or amplification at surgery, after neoadjuvant treatment^[11]^. This phenomenon is of particular interest in the landscape of breast cancer care because of its potential impact on treatment choice and prognosis. Although it has been reported in many studies, the reasons behind HER2 loss are still unclear. Both true biological changes and technical pitfalls have been assumed as potential causes of this phenomenon^[12]^ [Figure 1].

## BIOLOGICAL MECHANISMS DRIVING HER2 LOSS

Several biological mechanisms may be potentially responsible for HER2 loss, including HER2 downregulation, subtype switching, and clonal selection.

### HER2 downregulation

HER2 downregulation - defined as a reduction in HER2 expression at the proteomic level while retaining the *ERBB2* gene amplification - can be induced by anti-HER2 agents acting on the HER2 pathway itself^[14]^. In untreated HER2-positive tumors, cancer growth is driven by *HER2* gene amplification, which increases transmembrane HER2 tyrosine kinase (TK) receptor expression by up to 100-fold^[15]^. HER2 overexpression thus induces both homo- and hetero-dimerization of HER receptors, i.e., HER1, HER2, HER3, and HER4; phosphorylation of the kinase domains; and consequent activation of a downstream signaling pathway involving both the MAPK and PI3K/AKT/mTOR cascades^[16]^. 

Anti-HER2 drugs targeting the extracellular domain of HER2 have been shown to induce HER2 internalization by downregulating HER2 membrane expression in cells that retain HER2 amplification^[14]^. This phenomenon was first described in the early 2000s in cell cultures exposed to trastuzumab^[2,3]^, although it has not been confirmed by other studies^[14,17]^. To date, there is no clear explanation for these contradictory results. Some authors have suggested that immune cells may play a crucial role, since HER2-downregulation seems to occur only when trastuzumab is actively engaged with immune cells^[18]^. For instance, Shi and colleague observed HER2 downregulation only in co-cultures of cancer cell lines with immune cells and not in culture of cancer cells with trastuzumab only^[18]^. In particular, they detected an increased level of interferon gamma (IFN-γ) production by immune cells, which in turn activated the signal transducer and activator of transcription 1 (STAT1) that has been assumed to be responsible for HER2 transcriptional downregulation^[18,19]^. Indeed, the authors found an increased level of STAT1 in cancer cells with HER2 downregulation, whereas inhibition of STAT1 with fludarabine blocked the immune-related HER2 downregulation^[18]^. 

More recently, Bon and colleagues reported an even higher HER2 downregulation with the dual blockade by trastuzumab-pertuzumab combination^[20]^. The authors first observed a reduction in HER2 expression in cell lines after treatment with dual blockade and then demonstrated this *in vivo* by assessing HER2 in four patients with paired biopsies collected before and after exposure to trastuzumab-pertuzumab. HER2 downregulation at the membrane level was observed in all patients, although gene amplification by fluorescence in situ hybridization (FISH) was preserved in two of them^[20]^. Similarly, HER2-downregulation was observed after treatment with trastuzumab emtansine (T-DM1)^[21-23]^ and has been hypothesized as a potential mechanism of primary or secondary resistance to other anti-HER2 antibody-drug conjugates (ADCs)^[22,24]^. Of note, the recently presented correlative analysis from the DAISY study, which investigated trastuzumab deruxtecan in patients with HER2-positive, HER2-low (1+ or 2+/FISH-negative) and HER2 0 at IHC, showed a decrease of HER2 expression at progression in 65% of patients^[25]^. 

In contrast, TK inhibitors (TKIs), which act at the intracellular level, do not induce HER2 internalization but instead may increase HER2 expression by inhibiting HER2 phosphorylation and preventing receptor ubiquitination and internalization^[17]^. Indeed, treatment with TKIs might actually result in a marked accumulation of inactive receptors at the cell surface^[17]^. This phenomenon explains the rationale for combining TKIs with anti-HER2 antibodies, as their antitumoral activity is stronger in combination therapies rather than when single agents are administered^[17,26,27]^.

### Intratumor HER2 heterogeneity and clonal selection

Breast cancer is a highly heterogeneous disease, with differences in biology, gene expression profiles, and treatment sensitivity often coexisting in the same tumor area^[1]^. The exposure to anti-cancer drugs affects these cells differently, killing some cell populations and clonally selecting others. 

HER2 heterogeneity can thus result in HER2 loss due to treatment exposure, with HER2-positive subpopulations killed by treatment and resistant HER2-negative cells surviving. Many studies have characterized HER2 heterogeneity in breast cancer using various techniques, but very few of them have examined the correlation with HER2 loss^[1,28-30]^. 

In 2009, the ASCO/CAP guidelines defined HER2 heterogeneity as the presence of ≥ 5% to < 50% tumor cells with a ratio ≥ 2.2 when using dual probes or ≥ 6 HER2 signals/cell when using single probes^[31]^. Two to 4 representative areas of the invasive tumor should be selected and assessed after scanning the entire slide to look for heterogeneity. In 2013, this definition was updated by changing the cut-off value from 5% to 10% and the ratio from 2.2 to 2.0, in line with the updated definition for HER2-positivity^[32]^. Filho and colleagues applied the first definition to assess HER2 heterogeneity in two different areas of pretreatment biopsies of a cohort of 164 patients enrolled in a single-arm neoadjuvant trial administering T-DM1 plus pertuzumab and its impact on outcomes^[28]^. Of note, 16 out of 157 evaluable samples were classified as heterogeneous (10%), and none of them achieved a pathologic complete response (pCR) compared to 55% of non-heterogeneous tumors that achieved pCR^[28]^. Most of the heterogeneous cases were 2+ at IHC (75%) and ER-positive (81%). The authors also assessed heterogeneity as a fraction of the proportion of HER2 amplified cells defined by single-cell FISH and found an even higher correlation with non-pCR. Unfortunately, HER2 status on surgical samples and dynamics of heterogeneity were not reported^[28]^. 

Caswell-Jin and co-workers instead applied a whole-exome sequencing approach to evaluate HER2 heterogeneity in pretreatment samples and in different regions of surgical specimens after neoadjuvant anti-HER2 therapy in 5 patients^[33]^. By comparing the frequency of mutations across spatially distinct regions, they observed clonal replacement in two of five tumors, with mutations detected in surgical specimen that were absent in the pretreatment core biopsy. This suggests selection of resistant cells by neoadjuvant treatment in heterogeneous tumors, although it was not investigated whether this finding correlates with a different receptor profile across heterogeneous areas and HER2 loss was not investigated^[33]^.

### Subtype switch

According to gene expression profiles, breast tumors are classified into four major subtypes: luminal A, luminal B, HER2-enriched (HER2-E), and basal type^[34-36]^. Approximately 50%-60% of HER2-positive breast cancer are classified as HER2-E, with the remaining half distributed among luminal A (15%-20%), luminal B (10%-15%), and basal-like (5%-10%)^[37]^. HER2-E tumors are characterized by high expression of growth factor receptor-related genes (*ERBB2*, *EGFR*, and/or *FGFR4*) and cell cycle-related genes and low expression of estrogen-related and basal-related genes^[38]^. These tumor subtypes were initially characterized using microarray analysis evaluating more than 400 genes^[35,39]^, although the PAM50 classifier is currently applied for tumor subtyping in most correlative studies^[34]^. 

In a number of studies, platforms for gene expression profiling have been used to assess the tumor intrinsic subtypes of HER2+ BC both before and after neoadjuvant treatment^[40-43] ^[Table 1]. Of note, a subtype switch was observed in a substantial proportion of patients across different drug regimens, with the HER2-E to luminal A switch being the most frequent^[40-43]^. This finding could be attributed to a decreased expression of genes involved in cell proliferation after treatment exposure. Of note, gene expression analyses cannot distinguish among intra-tumor heterogeneity, stromal alterations, or a true treatment effect, so that stromal contamination might have a role in subtyping after treatment exposure. Interestingly, Brasó-Maristany and colleagues observed that this subtype switch can be reversible after anti-HER2 therapy discontinuation^[41]^.

**Table 1:** Neoadjuvant trials evaluating subtype switch during or after anti-HER2-based therapy

**Clinical trial**	**Patients (*n*)**	**Treatment**	**Paired samples (*n*)**	**Subtype switch**
**NeoSphere** Bianchini *et al.*^[42]^ 2018	417	HD, HPD, HP, PD	166	Significant increase in LumA and decrease in HER2-E and LumB subtypes from baseline to surgery
**PAMELA** Llombart-Cussac *et al.*^[43]^ 2017	151	HL	57	Baseline: HER2-E 67%, LumA 15%, basal-like 6%, LumB 10% normal-like 2%, D14: normal-like 49%, LumA 25%, HER2-E 18%, basal-like 6%, LumB 3%
**CALGB 40601** Carey *et al.*^[40]^ 2016	305	THL, TH, TL	78	Baseline*: HER2-E 15.4% LumA 39.7%, LumB 33.3%, basal-like 5.1%, claudin low 3.8%, normal like 2.6% Residual disease: HER2-E 8.9%, Lum A 48.7%, LumB 5.1%, basal-like 3.8%, claudin low 3.8%, normal-like 2.9%

H: Trastuzumab; P: pertuzumab; D: docetaxel; T: paclitaxel; *considering only patients with residual disease and paired analysis.

To our knowledge, none of these correlative studies performed in the context of neoadjuvant clinical trials correlated the subtype switch with HER2 loss on surgery specimens. These data were reported only by Pernas *et al*. in a retrospective study examining the PAM50 subtype in a cohort of 26 HER2-positive BC with paired samples and residual disease (RD) at surgery^[44]^. In this cohort, most HER2-E tumors (81.8%) converted to non-HER2-enriched, and a conversion to HER2-negative in residual disease was observed in 7/26 patients^[44]^.

## TECHNICAL PITFALLS

HER2 status assessment by IHC and ISH represents the standard of care for clinical decision making^[8]^. Thus far, these are the only two assays that proved to predict response to anti-HER2 therapy in several randomized clinical trials, therefore having solid clinical utility. IHC uses an antibody to assess HER2 protein expression, whereas ISH determines *HER2* copy number using a single probe for copies calculation or a dual probe that includes chromosome 17 centromere probe (CEP17) hybridization to determine the *HER2*/CEP17 ratio^[45]^. Different categories of ISH-based assays are available, including FISH or bright field-based techniques such as silver (SISH) or chromogenic in situ hybridization (CISH). Although FISH is considered the gold standard and is more used worldwide, SISH and CISH have shown very good concordance with FISH and can be considered as an alternative^[8]^.

Both IHC and ISH have shown a good concordance in assessing HER2 status, but standardization of laboratory testing - including accuracy, reproducibility, and precision - is needed, as technical variabilities can account for both false-negative and -positive results. In particular, ICH is highly dependent on tissue fixation methods, so that variable fixation time and different antibody clones and antigen retrieval methods can lead to incorrect IHC results. Conversely, ISH is less dependent on tissue fixation methods and more reproducible^[46,47]^, although other pitfalls can cause false results such as intratumoral heterogeneity, accidental assessment of in situ rather than invasive lesions, or suboptimal resolution (nonuniform signals, high autofluorescence, poor nuclear resolution, or high background-obscuring signal resolution)^[48]^. Additionally, ISH assessment on unstained sections stored for prolonged periods can be falsely read as negative. ASCO/CAP guidelines strongly recommend that laboratories performing HER2 testing should participate in regular laboratory inspections and biannual proficiency testing, such as the program offered by CAP^[8,32]^.

The technical variability of these assays accounts for the limited reproducibility of HER2 status across laboratories. Discrepancies in HER2-status between local and central testing have thus been demonstrated in many studies^[49-52]^. For instance, an analysis of the N9831 intergroup adjuvant trial identified a discordance rate of 18% and 12% for ICH and FISH, respectively, when comparing local and central laboratories, although a high degree of concordance was observed between central and reference laboratories^[53]^. The meta-analysis by Schrijver *et al*. evaluated instead receptor conversion from primary tumors to distant metastasis and showed a higher rate of discrepancy when HER2 was assessed with IHC (20.8%) instead of FISH (16.3%)^[10]^.

In 2018, the new ASCO/CAP guidelines introduced a 5-group classification for dual-probe ISH results to address specific scenarios of non-univocal interpretation, identifying for all these groups what additional work-up should be performed to obtain the most accurate classification of HER2 status. Groups 1 (HER2/CEP17 ratio ≥ 2, HER2 copy number > 4) and 5 (HER2/CEP17 ratio < 2, HER2 copy number < 4) identify the most frequent and straightforward categories of HER2-positive and -negative samples, respectively, whereas Groups 2-4 include the 5% of cases with a doubtful attribution. These cases frequently stain 2+ by IHC and are referred for a second opinion from a blinded pathologist. 

Amplification of *CEP17* represents one of the potential pitfalls causing dual-probe ISH test to fall into these groups. Specifically, cells with co-amplification of *CEP17* and *HER2 *are usually characterized by a HER2/CEP17 ratio < 2.0 and an average HER2 signals/cell ≥ 6.0, falling into Group 3. According to the 2018 ASCO/CAP guidelines, these samples should be reported as HER2-positive if IHC 2+/3+ is detected, although it was acknowledged that data on response to anti-HER2 therapy are limited. *CEP17 *gains or losses without involvement of the *HER2 *gene have also been reported, which lead to an under- or overestimation of HER2 amplification^[54]^. Of note, multiple gene amplifications on chromosome 17 may potentially involve further telomeric genes alteration (*TOP2*, *RARA*, *GRB7*, and *STARD3*), and this demands careful evaluation of the *HER2 *amplicon along with the potentially co-amplified neighboring genes, as well as possibly additional IHC assays for HER2^[55]^.

Further bias in HER2 assessment may result from sampling errors^[56]^. In the case of metastatic recurrence, guidelines recommend repeating HER2 testing in a metastatic site, if tissue sample is available, although a new biopsy cannot always be feasible^[8]^. Additionally, false-negative results can be caused by technical pitfalls, as frequently occurs in bone lesions, or by sampling bias due to intratumor heterogeneity^[57]^.

To improve diagnostic precision, some authors recommend also performing ISH in HER2-positive scored as 3+ or 0/1+ at IHC to confirm HER2 status^[58]^.The repeat biopsy, however, is not recommended for receptor re-evaluation, as it does not improve diagnostic accuracy^[49]^. 

More recently, HER2 assessment on liquid biopsies has emerged as potential alternative to tissue sampling. Possible sources of HER2 status assessment are circulating tumor cells, cell-free tumor DNA (ctDNA), and extracellular vesicles. The advantage of the method is low invasiveness, albeit copy number assessment is complicated due to the vast background of healthy material, tumor heterogeneity, and high signal-to-noise ratio. Thus, HER2 assessment on liquid biopsies remains investigational and should not be recommended for clinical decision making^[57]^. 

## CONCLUSION

Loss of HER2 is not a rare phenomenon, being detected in up to 50% of breast tumors in different settings. Several mechanisms may contribute to this finding, including intratumor heterogeneity, clonal selection, and true biological modifications such as HER2 downregulation or subtype switch. Nevertheless, these associations are mainly speculative, as no study has specifically linked these phenomena to HER2 loss in tissue specimens. On the other side, analytical pitfalls may be responsible for some false-negative results, and some authors have suggested that technical issues are responsible for most cases of HER2 loss. The limited reproducibility of HER2 retesting across laboratories is well-known and can exemplify the technical variability behind these tests^[49]^, especially in immunohistochemical evaluation. In contrast, assessment of gene amplification by ISH is less dependent on technical variables. The ESMO Clinical Practice Guidelines for diagnosis, treatment, and follow-up of primary breast cancer suggest that HER2 gene amplification status may be determined directly from all invasive tumors using any type of in situ hybridization (fluorescent, chromogenic, or silver), completely replacing IHC or only for tumors with an ambiguous (2+) IHC score (II, B)^[59]^. 

Many studies investigated receptor conversion both in the neoadjuvant setting and at metastatic relapse^[9,11,60,61]^. Nevertheless, most of them were small, retrospective, included patients receiving different drug regimens, and applied distinct definitions of HER2-positivity, thus leading to many biases and low statistical power. Additionally, most studies were conducted before the approval of trastuzumab in the (neo)adjuvant settings, so most patients did not receive anti-HER2 therapy^[62]^. Notably, none of these studies performed correlative analyses to understand the biology behind HER2 loss, but some of them investigated whether analytical problems may have been responsible for some of these cases. When also adding FISH analysis in IHC 0 or 1+ cases, some authors disproved cases of HER2 loss^[20,61-64]^. This is particularly relevant after administration of trastuzumab or other agents targeting the HER2 extracellular domain, due to the potential downregulation of membrane expression with persisting gene amplification^[14,20]^. Interestingly, this phenomenon may be reversible, so that HER2 loss may be falsely detected if samples are collected during targeted therapy^[41,65]^. Although is not possible to distinguish reversible from definitive HER2 loss, it might be argued that patients retaining HER2 amplification at ISH are more prone to restore HER2 expression after stopping anti-HER2 therapy. Unfortunately, timing of HER2 re-overexpression for cases with transient HER2 loss is unknown. To minimize the chance of false-negative results, we believe ISH testing should be performed in all cases of HER2 loss, even in tumors scored as 0 or 1+ at IHC. Indeed, ISH can detect gene amplifications not only in cases of HER2 downregulation but also when technical artifacts lead to a false negative result^[49]^. Given the efficacy of new anti-HER2 therapies, determination of HER2 addiction/targetability is crucial and may open important therapeutic options with potential impact on survival.

In conclusion, many studies have described HER2 loss in various settings, but correlative studies specifically addressing this phenomenon, i.e., studies collecting and analyzing samples from patients with HER2 loss at different timepoints (before, during, and after therapy), are lacking, and the extent to which technical pitfalls contribute to HER2 loss is unknown.

We believe translational studies that match clinicopathological and biologic features are warranted, to both shed light on this phenomenon and provide guidance on which drugs and strategies may be more effective for patients with HER2 loss. 

## DECLARATIONS

### Copyright

© The Author(s) 2022.
